# Formulation and Characterization of Rifampicin Microcapsules

**DOI:** 10.4103/0250-474X.62240

**Published:** 2010

**Authors:** MD. Sarfaraz, D. Hiremath, K. P. R. Chowdary

**Affiliations:** Department of Pharmaceutics, N. E. T. College of Pharmacy, Raichur-584 101, India; 1 Industrial Pharmacy Division, University College of Pharmaceutical Sciences, Andhra University, Visakhapatnam-530 003, India

**Keywords:** Carbopol 974P (CP), microcapsule, rifampicin, Sodium Alginate (SA)

## Abstract

Rifampicin biodegradable microcapsules were prepared by feasible emulsification-ionic gelation method for a novel controlled release product. Sodium alginate and Carbopol 974P were used as coating polymers in different ratios 1:1, 1:2, 1:3 and 1:4 to obtain elegant microcapsules. The formulations were characterized for encapsulation efficiency, drug loading, sieve analysis, scanning electron microscopy and *in vitro* release studies. The microcapsules were discrete, large, almost spherical and free flowing with encapsulation efficiency in the range of 75% to 89%, drug loading 75% to 86% and size 440 μm to 500 μm. Rifampicin release from these microcapsules was slow and extended over longer periods of time depending on the polymer coat. Drug release was diffusion controlled and followed first order kinetics. The formulation MC1 with a coating ratio of 1:1 (Sodium alginate: Carbopol 974P) was found to be suitable for oral controlled release.

Controlled release dosage forms are becoming increasingly important, either to achieve the desired level of therapeutic activity required for a new drug entity or to extend life cycle of an existing drug through improved performance or patient compliance. Microsphere technology is one of the established techniques for controlled delivery used to deliver several different types of drugs, such as steroids[1], peptides[2], proteins[3], and antibiotics[4,5]. The polymers used are biodegradable by means of nonenzymatic degradation and can be formulated for release up to several months or years, depending on the chemical and physical properties of the specific drug to be encapsulated and the specific polymeric excipient that will be used. The purpose of this investigation was to develop microcapsules utilizing blend of polymers for delivery of antimycobacterial drug.

Rifampicin, a semi synthetic hydrazine derivative of rifampicin B was chosen for the study because it is one of the established first-line drugs used to treat tuberculosis and its biological half-life varies from 1.5 to 5 h.

Carbopol 974P is a synthetic high molecular weight cross-linked polymer of acrylic acid. It is a safe and nontoxic. It is an excellent thickening, emulsifying, suspending and gelling agent[6]. Because of its control release and gel formation properties, Carbopol 974P was chosen as the encapsulation polymer.

Alginates are a family of polysaccharides composed of α-L-glucoronic acid (G) and β-D-mannuronic acid (M) residues, arranged in homopolymeric blocks of each type (MM, GG) and in heteropolymeric blocks of each composition. Alginates form strong gels with divalent cations like Ca^2+^, giving both strength and flexibility. Such a cross-linking process stiffens and roughens the polymer and reduces the swelling in solvents. The soluble sodium alginate was cross-linked with calcium chloride, resulting in the formation of the insoluble calcium alginate. This strong thermo stable gel has properties that largely depend on the characteristics of the polymer and the preparation method[7–9].

Rifampicin was obtained as gift sample from On Top Pharmaceuticals (Bangalore, India). Carbopol 974P was obtained from Astra Zeneca Labs. (Bangalore, India). Sodium alginate was procured from Rolex Laboratories (Bangalore, India). Tween-80, ascorbic acid LR and calcium chloride were purchased from S. D. Fine Chemicals (Mumbai, India). Toluene and petroleum ether were purchased from Nice Chemicals (Cochin, India), while castor oil was procured from Standard Labs. (Hyderabad, India). All other reagents used were of analytical grade.

Microcapsules containing rifampicin were prepared employing carbopol 974P in combination with sodium alginate as coat materials. No methods are reported for micro encapsulation by these polymers. The ionic gelation process[10,11], which has been used to prepare large sized alginate beads, was used to prepare the microcapsules. Carbopol 974P and sodium alginate in different ratios (Table 1) were soaked in 1% w/v Tween-80 (10 ml) and in purified water (5 ml) respectively, for overnight to form a homogenous polymer solution. The two polymers were uniformly mixed with the help of a stirrer. The core material rifampicin (0.3 g) was dissolved in methanol (1 ml) and added to the polymer solution and mixed thoroughly to form a smooth viscous dispersion. The resulting dispersion was then added in a thin stream with syringe to about 250 ml of castor oil contained in a 500 ml beaker, while stirring at 250±20 rpm. A Remi medium duty overhead stirrer with speed meter (Model RQT 124) was used for stirring. The stirring was continuous for 5 min to emulsify the added dispersion as fine droplets. Calcium chloride (20% w/v) solution (40 ml) was then added slowly while stirring for ionic gelation (or curing) reaction to occur. Stirring was continued for 20 min to complete the curing reaction and to produce spherical microcapsules. The microcapsules so obtained were filtered and washed thrice with petroleum ether (60 ml). Later they were dried in air for 3 h followed by hot air oven at 50° for 4 h and stored in a dessicator.

**TABLE 1:** CHRACTERISTICS OF THE PREPARED MICROCAPSULES

Microcapsules	Coat composition	Rifampicin content (%)*	Micro encapsulation efficiency (%)	First order model r	M_t_/M_α_ = Kt^n^ n
Size-22/44					
MC 1	SA - CP (1:1)	12.79±0.15	89.84	−0.995	0.423
MC 2	SA - CP (1:2)	10.81±0.27	75.70	−0.996	0.482
MC 3	SA - CP (1:3)	17.20± 0.15	86.00	−0.998	0.477
MC 4	SA - CP (1:4)	13.24± 0.27	79.47	−0.988	0.293

Coat composition, rifampicin content, microencapsulation efficiency and release kinetics of the prepared microcapsules.

* Figures in parenthesis are expressed as mean±standard deviation of 3 observations. MC represents microcapsule while SA is sodium alginate and CP is Carbopol 974P.

Size of the rifampicin microcapsules was determined by sieve analysis. The microcapsules were separated in to different size fractions by sieving using standard sieves. The particle size distributions of the microcapsules were determined to know the mean particle size of microcapsules. All readings are average of three trials ±SD.

The shape and surface morphology of the rifampicin microcapsules were investigated using a Hitachi-S5-20, scanning electron microscope at room temperature using the required magnification. The sample was deposited on alluminium stubs and coated with gold in HUS-5GB vacuum evaporator, to render it electrically conductive. Encapsulation efficiency was calculated using the formula, Encapsulation efficiency= (estimated percent drug content/theoretical percent drug content)×100.

Rifampicin content in the microcapsules was estimated by using UV spectrophotometer method. A quantity of microcapsules equivalent to 100 mg were powdered and transferred into a 100 ml volumetric flask, sufficient amount of methanol was added to produce 100 ml, shaken for 20 min and filtered. Two milliliters of the filtrate was diluted to 100 ml with phosphate buffer (pH 7.4) containing ascorbic acid (200 μg/ml) and the drug content was analyzed at 475 nm[12]. Three determinations were carried out for each fraction.

Dissolution studies were carried out using USP XXIV rotating basket method. The stirring rate was 75 rpm. 900 ml of pH 7.4 phosphate buffer having ascorbic acid (200 μg/ml) was used as dissolution medium and maintained at 37±1°. Samples of 5 ml each were withdrawn at regular time intervals, filtered, diluted suitably, analyzed using double beam UV spectrophotometer at 475 nm and an equal volume of fresh medium was immediately added to maintain the dissolution volume. Dissolution studies were carried out up to 12 h. The drug release experiments were conducted in triplicate.

The results obtained were fitted according to the following exponential equation, M_t_/M_α_ = Kt^n^, where M_t_/M_α_ is the fractional solvent absorbed or drug release at time ‘t’; K denotes a constant incorporating the properties of the macromolecular polymeric system and the drug and ‘n’ is a kinetic constant which depends on time and is used to characterize the transport mechanism. While n= 0.45 for case I or Fickian diffusion which is characterized by a square root time dependence in both the amount diffused and the penetrating diffusion front position; n= 0.89 for case II transport, which is completely governed by the rate of polymer relaxation, exhibits a linear time dependence in both the amount diffused and the penetrating swelling front position; n= 0.45<n<0.89 for anomalous behavior or non-Fickian diffusion and polymer relaxation are comparable[13].

IR studies (not shown) indicated that the qualitative composition of the drug formulation and verified the identity of each of the components. No drug interaction or complexation occurred during the manufacturing process.

Kockisch *et al,* have prepared carbopol microspheres by using modified U-shaped beaker, a two blade paddle stirrer and maintaining a temperature of 60° for a period of 24 h to promote the evaporation of water[14,15]. Microcapsules of rifampicin with carbopol alone as a coat were difficult to prepare, perhaps due to inadequate plasticity, which may be attributed to the fact that calcium chloride competes with carbopol for water molecules, thereby decreasing the hydration of carbopol[16,17]. Hence in the present research microcapsules with a coat consisting of carbopol 974P and sodium alginate in different ratios were prepared by an emulsification and ionic-gelation process. The concentration of sodium alginate was kept constant and the carbopol concentration was increased with each formulation (Table 1). The microcapsules were found to be almost spherical, discrete, and free flowing. Microphotographs (fig. 1) indicated that the microcapsules were also almost spherical and surface was not smooth but contained little cracks and deposits. The sizes could be separated and more uniform size range of microcapsules could readily be obtained. The sieve analysis of different microcapsules (Table 2) showed that a large proportion of microcapsules (60-75%) in a batch were in the size range of −22 +44 (468 μm) mesh. A log-normal size distribution of the microcapsules was observed in all batches prepared. Low SD values in percent drug content indicated uniformity of drug content in each batch of microcapsules (Table 1). The microcapsulation efficiency was in the range (76-90%). Drug content of the microcapsules was found to be the same in different sieve fractions.

**Fig. 1:**
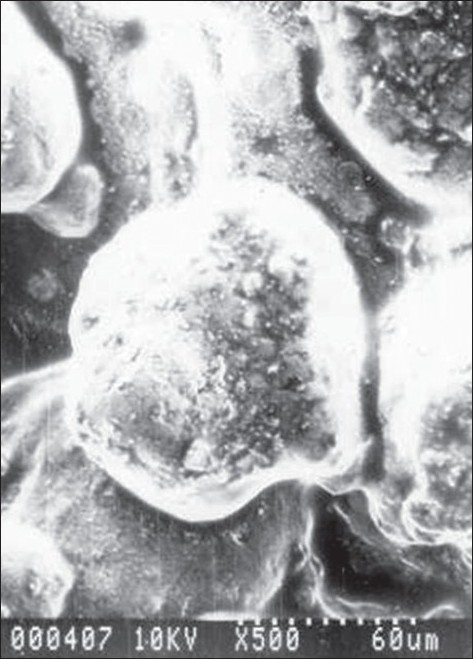
SEM image of rifampicin-loaded microcapsules. Scanning electron micrographs of rifampicin-loaded microcapsules (MC1) prepared by ionic gelation process taken at scope magnifications of ×500.

**TABLE 2:** PARTICLE SIZE OF PREPARED RIFAMPICIN MICROCAPSULES

	Particle size (μm)^a^
MC 1	444.79±4.22
MC 2	467.43±5.07
MC 3	486.52±3.73
MC 4	501.77±2.89

a Mean±SD

Rifampicin release from the microcapsules was studied in phosphate buffer pH 7.4 containing ascorbic acid (200 μ/ml) in dissolution test apparatus USP XXIV using rotating basket. Rifampicin release from the microcapsules was slow and spread over a period of 12 h and depended on coating ratio (SA:CP= 1:1, 1:2, 1:3, 1:4) and size of the microcapsules. As the proportion of carbopol 974P was increased, rifampicin release rate was decreased from 91.2, 81.7, and 80.5 to 69.5% respectively.

In order to understand the mechanism of drug release, the experimental data was fitted to the exponential equation[13]. The linear correlation coefficient of the slopes and slope values of the graphs when drawn the graph between Log (M_t_/M_α_) versus Log t (Table 1), indicate that the release kinetics conform predominantly to case I diffusion (n= 0.48). The classical Higuchi type of release mechanism can be explained as a result of the rapid hydration of the polymer molecules on the surface of the microcapsules, which results in a gel or a light viscous solution surrounding the microcapsules that restricts water penetration in to the centre. The net result is a reduction of the rate of the drug release as a function of time. It can be observed from the data the linear correlation coefficients of the first order model when drawn the graph between Log percent of drug retained Vs time provided an adequate fit (r>0.98) to the release profile of the microcapsules[18].

Carbopol 974P due to hydration swells and forms a gel like layer around the microcapsule and release the drug at a reduced rate as a function of time (MC4). If the polymer does not hydrate quickly the surface barrier or gel is not formed immediately, it results in a large portion of drug to be released during the initial phase of release profile (MC1) (fig. 2). Sodium alginate due to cross linking with calcium chloride forms a tight junction between the guluronic acid residues. The release of drug from calcium-alginate-carbopol microspheres is also due to the rapid removal of calcium as calcium phosphate from the microspheres due to ion-exchange process with sodium ions of phosphate buffer medium[19].

**Fig. 2:**
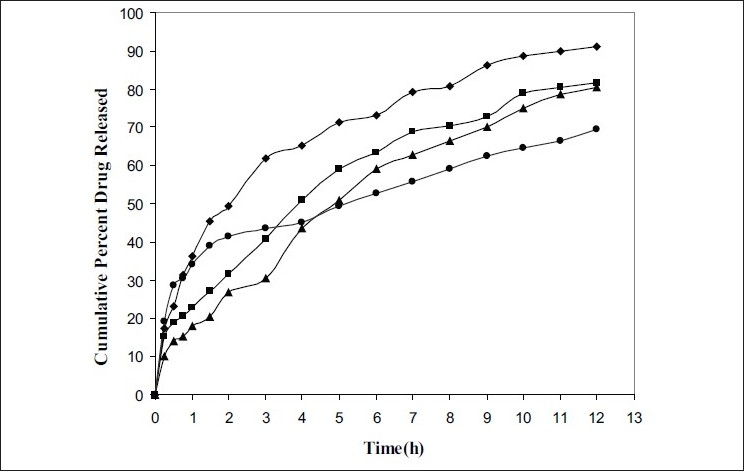
Dissolution profiles of carbopol-alginate microcapsules of rifampicin. *In vitro* dissolution studies of rifampicin microcapsules in pH 7.4 phosphate buffer at 37°, MC I (–♦–), MC II (–■–), MC III (–▲–) and MC IV (–●–).

Spherical microcapsules with a coat consisting of alginate and carbopol could be prepared by emulsification-ionic gelation process. Microencapsulation efficiency was found to be in the range 76-90%. Rifampicin release from the polymer coated microcapsules was slow and extended over longer periods of time and depended on core: coat ratio and size of microcapsules. Drug release from the polymer coated microcapsules was by fickian diffusion and followed first order kinetics. Thus carbopol 974P and sodium alginate coated rifampicin microcapsules were found suitable for oral controlled release products.
